# Occurrence of Bisphenols and Benzophenone UV Filters in White-Tailed Eagles (*Haliaeetus albicilla*) from Smøla, Norway

**DOI:** 10.3390/toxics9020034

**Published:** 2021-02-09

**Authors:** Bernat Oró-Nolla, Silvia Lacorte, Kristine Vike-Jonas, Susana V. Gonzalez, Torgeir Nygård, Alexandros G. Asimakopoulos, Veerle L.B. Jaspers

**Affiliations:** 1 Department of Environmental Chemistry, IDAEA-CSIC, Jordi Girona 18-26, 08034 Barcelona, Catalonia, Spain; bonqam@cid.csic.es (B.O.-N.); silvia.lacorte@idaea.csic.es (S.L.); 2 Department of Chemistry, Norwegian University of Science and Technology (NTNU), 7491 Trondheim, Norway; kristine.vike@ntnu.no (K.V.-J.); susana.v.gonzalez@ntnu.no (S.V.G.); alexandros.asimakopoulos@ntnu.no (A.G.A.); 3 Department of Biology, Norwegian University of Science and Technology (NTNU), 7491 Trondheim, Norway; 4 Norwegian Institute for Nature Research (NINA), Høgskoleringen 9, 7034 Trondheim, Norway; Torgeir.Nygard@nina.no

**Keywords:** emerging contaminants, bisphenols, benzophenone UV filters, raptor biomonitoring, white-tailed eagle, liver

## Abstract

There is a growing concern about the occurrence of bisphenols and benzophenone UV filters in natural ecosystems, while data are limited regarding their actual occurrence in wildlife species, especially in raptors. In this study, concentrations of bisphenol and benzophenone UV filter analogues were determined in liver tissue samples (*n* = 38) from white-tailed eagles (*Haliaeetus albicilla*) that were found dead in Smøla (2006–2018), which is a Norwegian municipality that holds one of the densest breeding populations of white-tailed eagles in Europe. Bisphenol AF (BPAF; a fluorinated analogue) was the most ubiquitous contaminant since it was detected in 32 liver samples at concentrations ranging from 1.08 to 6.68 ng/g wet weight (w.w.), followed by bisphenol A (BPA, mean 10.4 ng/g w.w.), benzophenone-1 (BzP-1, mean 3.24 ng/g w.w.), and 4-hydroxybenzophenone (4-OH-BzP, mean 0.62 ng/g w.w.). The concentrations found in livers suggested that white-tailed eagles potentially accumulate bisphenols and benzophenone UV filters, which raises concern, as these plastic and personal care product-related emerging contaminants can show endocrine-disrupting properties. The high detection frequency of the fluorinated BPAF warrants further attention as other fluorinated compounds have proven to be extremely persistent and potentially harmful to wildlife.

## 1. Introduction

Raptors (owls and birds of prey) are sensitive to pollution pressures as they bioaccumulate high concentrations of contaminants due to their apex trophic position [1,2,3]. Biomonitoring contaminants in raptors has emerged as a promising approach to reflect pollution pressures in the environment [4]. Historically, the biomonitoring of persistent organic pollutants (POPs) in raptors was initiated when it was first reported that 4,4′-dichlorodiphenyltrichloroethane (DDT) exposure decreased the thickness of eggshells and led to raptor population declines [5]. Nowadays, numerous studies report bioaccumulation of legacy POPs in raptors in Europe: e.g., polychlorinated biphenyls (PCBs) and organochlorinated pesticides (OCPs) in white-tailed eagles (*Haliaeetus albicilla*) [6]; PCBs in kites (*Milvus milvus*) [7]; polybrominated diphenyl ethers (PBDEs) in barn owls (*Tyto alba*) [8]; and organophosphate ester flame retardants (OPEs) in cinerous vultures (*Aegypius monachus*) [9]. Recently, per- and poly-fluoroalkyl substances (PFASs) were also reported as emerging POPs in barn owls, white-tailed eagles, and northern goshawks (*Accipiter nisus*) [10,11,12,13]. However, there is little information regarding the accumulation of emerging contaminants in raptors.

Currently, there is a growing concern over the occurrence of bisphenols (BPs) and benzophenone UV filters (BzPs) in the environment [14,15], since these contaminants are widespread in aquatic systems and can potentially accumulate in soil, sediments, and tissues of living organisms [16]. Bisphenol A (BPA) is the most known BP, since it is extensively used as a monomer for polycarbonate plastic and epoxy resin production [17]. However, a broad range of adverse effects are reported for BPA, classifying it as an endocrine-disrupting chemical [18,19]. Therefore, due to implemented restrictive regulations towards the use of BPA, other alternative BP analogues have been introduced to the global market [20,21,22]. Bisphenol F (BPF) is currently used in the production of low viscosity epoxy resins [23]. Bisphenol S (BPS) is found, among others, in paper products (e.g., mailing envelopes, thermal receipt papers), dyes and tanning agents [15,24]. Bisphenol AF (BPAF) is a fluorinated chemical commonly used as a cross-linking reagent in the production of fluoropolymers and fluoroelastomers and as a monomer in the production of other polymers, e.g., polyimides [15]. BPAF also has documented endocrine disruption effects, such as testosterone reduction [25]. An in vitro study suggested that BPA, BPF, BPS and BPAF can stress and damage human red blood cells, while BPAF demonstrated the strongest impact in altering antioxidant enzyme activity [26].

The second emerging pollutant class, BzPs, are mostly applied as ingredients in sunscreen products and cosmetics for topical application, but also as additives in plastics for photo-stabilization purposes [27]. The release of plastic products into the environment and the use of sunscreens are the main exposure sources of BzPs in aquatic ecosystems, and have been reported to potentially cause reproductive effects in fish [14].

In 2013, the Norwegian Environmental Agency (Miljødirektoratet) determined BPs and BzPs in marine biota in the Oslofjord and Lake Mjøsa (Norway). In the Oslofjord, BPs were detected in northern shrimps (*Pandalus borealis*, median concentrations: 0.06–19.7 ng/g w.w.) and cod liver (*Gadus morhua*, median concentrations: 1.00–590 ng/g w.w.), and occasionally in shore crabs (*Carcinus meanas*). In Lake Mjøsa, BPs were frequently detected in perch (*Perca fluviatilis*, median concentrations: 0.30–260 ng/g w.w.), whitefish (*Coregonus lavaretrus*, median concentrations: 0.30–250 ng/g w.w.), brown trout (*Salmo trutta*, median concentrations: 0.20–60.0 ng/g w.w.), and burbot liver (*Lota lota*, median concentrations: 1.00–18.0 ng/g w.w.). BzPs were found at trace concentrations in the Oslofjord with BzP-3 ranging from 20.0 to 1037 ng/g w.w. [28,29].

Nonetheless, relevant biomonitoring data regarding the occurrence and distribution of BPs and BzPs in raptors remains scarce. So far, only one peer-reviewed study has been published on BPs and BzPs in raptor tissues from France and Greenland [30], and there is a lack of data on other places in Europe, especially in Scandinavia. With this background, the present study aims to explore for the first time the occurrence of BPs and BzPs in raptors from Northern Europe. We studied a series of bisphenols, including the well-known bisphenol A, but also other bisphenols not as commonly reported in environmental matrices. These compounds may have high environmental impact because they are used in very large quantities and are suspected to be strong endocrine-disrupting compounds, and yet their occurrence in wildlife in Northern Europe is not studied. Thus, we analyzed liver tissue obtained from white-tailed eagle (*Haliaeetus albicilla*) carrions collected from Smøla municipality (Norway), which holds one of the densest breeding populations of white-tailed eagles in Europe.

## 2. Experimental Section

### 2.1. Chemicals and Materials

The target analytes studied here and their main physicochemical properties are listed in Table S1. Other relevant information was reported by González-Rubio et al. (2020) [30]. Standards of BPA (≥99%), BPAF (≥99%), bisphenol B (BPB, ≥98%), BPF (≥98%), BPS (≥98%), bisphenol M (BPM, ≥99%), bisphenol P (BPP, ≥99%), benzophenone-1 (BzP-1, ≥99%), benzophenone-2 (BzP-2, ≥97%), benzophenone-8 (BzP-8, ≥98%) and 4-hydroxybenzophenone (4-OH-BzP, ≥98%) were purchased from Sigma-Aldrich (Steinheim, Germany). Internal standards (ISs) were purchased from Cambridge Isotope Laboratories (Andover MA, USA): ^13^C-isotope of BPA (BPA-^13^C_12_; ≥99%), BPAF (BPAF-^13^C_12_; ≥99%), BPB (BPB-^13^C_12_; ≥99%), BPF (BPF-^13^C_12_; ≥99%), and BPS (BPS-^13^C_12_; ≥98%).

### 2.2. Study Population and Sample Collection

White-tailed eagles are large and long living raptors which base their diet on fish, but they can also eat marine birds that live near the shore or estuaries [31]. In 1988, these eagles were listed as a threatened species by the International Union for Conservation of Nature (IUCN) [32], but today they are listed as of least concern and are considered a good sentinel species to monitor environmental pollution [12].

**Figure 1:**
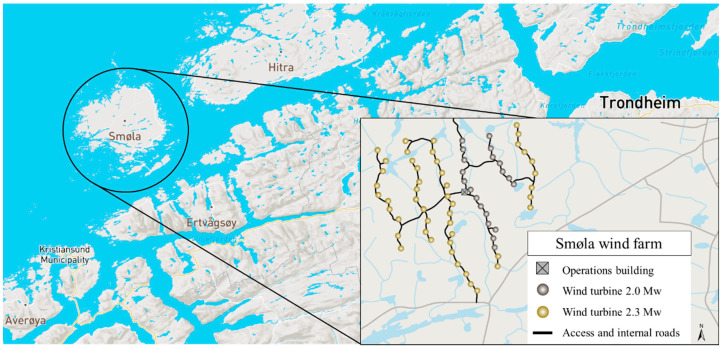
Location of Smøla island in Norway [36]; right corner: Focus on the Smøla wind farm; showing the web of turbines located on the island [34].

The sampling was conducted in Smøla (63°27′ N; 8°0′ E), a Norwegian municipality which holds one of the densest breeding population of white-tailed eagles in Europe, estimated at more than 50 breeding pairs [33]. This island and its archipelago are an excellent habitat for this species because it is flat and windy, and the food is abundant in the shallow sea surrounding it. In 2002, a wind farm was established in Smøla (Figure 1), which was at that time the largest on-land windfarm in Europe, with 68 turbines of 2–2.3 megawatts, benefiting from the excellent wind conditions of the location (Statkraft: Smøla wind farm) [34]. Unfortunately, this web of wind turbines became a problem for the white-tailed eagles since they are at risk of colliding with them. Breeding success and mortality after the construction of the wind-power plant was affected in a radius of 500 m from the turbines [33,35].

The white-tailed eagle tissues were obtained between 2006 and 2018 from victims of the wind turbine collisions in Smøla. A set of 38 individuals consisting of 21 adults (11 females and 10 males), 15 subadults (6 females and 9 males), 1 juvenile (male) and 1 nestling (male) were analyzed as they had intact livers when found (Table S2). The age and sex of the birds was determined by plumage characteristics such as the molt patterns of the contour feathers (wing and tail), the colour of the bill and tail, and full range of biometrics [37]. Tissues were carefully dissected using stainless steel tools rinsed thoroughly between tissues and individuals. All samples were properly labelled and stored in the darkness at −20 °C. Sampling was approved by the Norwegian Food Safety Authority (Mattilsynet, Oslo, Norway)—the Experimental Animal Committee (FOTS ID 6432, 6 May 2014).

### 2.3. Sample Preparation and Analysis

Liver samples of 0.1–0.2 g were placed into 15 mL polypropylene (PP) tubes and the target analytes were extracted from the liver matrix and analyzed using the protocol reported by González-Rubio et al. (2020) [30]. Briefly, the sample preparation method involved the incubation of the samples in ammonium acetate (1.0 M) with *β*-glucuronidase at 37 °C for 12 h to establish total concentrations (free and conjugated chemical species), followed by solid–liquid extraction with ethyl acetate. Instrumental analysis was performed by ultra-performance liquid chromatography tandem mass spectrometry (UPLC-MS/MS) using a Waters Xevo™ TQ-S Triple Quadrupole Mass Spectrometer from Waters Corporation (Milford, MA, USA). Further details about the analytical method and method performance can be found in supporting information.

### 2.4. Quality Assurance and Quality Control (QA/QC)

The performance of the method was monitored by analyzing non-spiked, pre-extraction spiked and post-extraction spiked in pooled samples of 5 white-tailed eagle livers. Potential contamination during sample preparation was evaluated by analyzing three reagent blanks that followed the whole procedure as actual samples. The analytical performance of the method was monitored through correlation coefficients, precision, instrumental and method detection (IDLs and MDLs) and quantification (IQLs and MQLs) limits, matrix effects (MEs %) and extraction recoveries (Tables S3–S5). Trueness of the method was assessed through relative recoveries (adjusted by ISs) in the liver by fortification of the target analytes (fortified amounts: 2.5, 10, 20 and 50 ng; *n* = 4 replicates for each amount with 10 ng ISs) spiked before extraction (pre-spiked) and post-extraction (in the final extract). The mean recoveries for BPs and BzPs are presented in Table 1. Procedural blanks revealed a contribution of ~5% of BPAF from the background, and consequently, these amounts were subtracted in the calculations of the recoveries and the final sample concentrations.

The IDLs and IQLs were estimated in the solvent matrix for every target analyte as 3 and 10 times the signal-to-noise ratio, respectively. Similarly, the MDLs and MQLs were estimated for every target analyte as 3 and 10 times the signal-to-noise ratio, respectively, by using a fortified liver sample with an amount of 2.5 ng (corrected for endogenous amounts). Estimation of MEs (%) during analysis was performed as described by González-Rubio et al. (2020) [30]. UPLC-MS/MS data were acquired with the MassLynx v4.1 software, while quantification processing was performed with TargetLynx (Waters, Milford, MA, USA). Data treatment did not involve non-detects and concentrations were reported as ng/g wet weight (w.w.).

## 3. Results and Discussion

The concentrations and detection rates of the detected BPs and BzPs in the liver of white-tailed eagles from Smøla, Norway are presented in Table 2. Among the target analytes, BPA, BPAF, 4-OH-BzP and BzP-1 were the most abundant in terms of concentration and frequency of detection, whereas BzP-2 and BzP-8 were found in a few samples only; other contaminants were not detected. The occurrence of BPs and BzPs in livers indicates that they can enter the blood circulation and subsequently accumulate in the liver of the bird.

### 3.1. Occurrence of BPs

Among the 38 samples analyzed, BPA was found in eight individuals at concentrations ranging from 3.36 to 33.8 ng/g w.w., and its mean concentration of 10.4 ng/g w.w. was the highest among the detected analytes. The highest BPA concentration (33.8 ng/g w.w.) was found in a single sample from an adult male, while the sub-adult males and females (Table S6) had lower concentrations. Mean concentrations were 13.9 and 6.86 ng/g w.w. for males and females, respectively, but their differences were not statistically significant (*p* > 0.05). Nonetheless, the tendency for lower concentrations in females can be potentially attributed to maternal transfer, which decreases the burden of contaminants in the mother and transfers it to the eggs and chicks [9,38]. Although restrictions are in place in Europe (including Norway) and the USA, BPA is currently being produced at a rate of 100,000–1,000,000 tons per year in Europe [39] and 1,000,000 tons per year in the USA [40]. In Norway, the Norwegian Environmental Agency reported that the BPA consumption was reduced from ~4.5 ton in 2007 to 0.5 ton in 2017, with a future perspective of stopping the emissions within 2020 [41]. However, in the current study, no significant changes were observed between years in BPA concentrations in livers of the white-tailed eagles that would correspond to BPA usage restrictions (Table S7). From the BPs class, only BPA has so far been studied extensively in bird livers. Staniszewska et al. (2014) [42] reported a BPA concentration of 324 ng/g dry weight (d.w.) in the liver (*n* = 1) of a great black-backed gull (*Larus marinus*) and a mean concentration of 111 ng/g d.w. in livers (*n* = 10) of herring gulls (*Larus argentatus*). Differences in feeding patterns and temporal or regional exposure, but also physiological differences of the species, could potentially explain pollutant concentration differences [42,43,44].

BPAF was the most ubiquitous compound in this study, with concentrations ranging from 1.08 to 6.68 ng/g w.w. in 32 out of the 38 samples. Mean concentrations were 2.90 and 2.44 ng/g w.w. for adult males and females, respectively, but their differences were not statistically significant (*p* > 0.05). The sub-adults (mean concentration: 3.15 ng/g w.w.) and the single juvenile individual (4.54 ng/g w.w.) demonstrated higher BPAF concentrations, which can potentially denote either maternal transfer from the egg or dietary accumulation at an early age (range: 1.70–6.68 ng/g w.w.) (Table S6). This pollutant was detected in all years (Table S7) with a mean concentration ranging from 1.88 ng/g w.w. in 2006 to 4.71 ng/g w.w. in 2009. Production and use of other BP analogues, such as BPAF and BPF, have increased in recent years [15] due to BPA regulations/restrictions. In particular, BPAF has been produced annually at a rate of 4.5–230 tons in the USA in 2008 [20], while nowadays it is produced at a rate of 100–1000 tons per year in Europe [45]. The production rise in Europe can explain the higher detection rate for BPAF in white-tailed eagles from Norway compared to the study of González-Rubio et al. (2020) [30], where the raptor samples were collected from Greenland before 2008 when the production in the USA was significantly lower. Although there are no data on the effects of BPAF in raptors, malformation and lower survival rate at an exposure concentration of 5 µg/L was reported in zebrafish offspring [46]. It is also reported that BPAF is more harmful, since its CF_3_ moiety is more reactive than the CH_3_ group of BPA [47]. Some fluorinated pollutants, e.g., perfluorooctanesulfonic acid (PFOS), are proven to be extremely persistent and harmful for living organisms [48], and therefore BPAF toxicity and occurrence should be further investigated.

BPF was not detected in this study, even though it was the most abundant BP in the liver of white-tailed eagles from West Greenland, with concentrations ranging from 1.22 to 7.32 ng/g w.w. [30]. The differences in the BPs profile between the Norwegian and Greenland white-tailed eagle populations can be attributed to the different sampling locations, the potential different usage in Europe versus North America, but also to the time-period of sampling. The Greenland samples were collected between 1997 and 2009, while the samples in the present study were collected between 2006 and 2018. To the best of our knowledge, no data of production or usage have been reported or provided for BPF according to ECHA (2020) [49], and in the literature this lack of information is clearly indicated [20,21,22].

### 3.2. Occurrence of BzPs

For the BzPs, BzP-1 was detected in 10 out of 38 samples at concentrations ranging from 2.07 to 7.94 ng/g w.w, followed by 4-OH-BzP, which was detected in eight samples at a range of 0.14 to 2.08 ng/g w.w. BzP-8 was detected in a sub-adult female (10.5 ng/g w.w.) and a sub-adult male (2.08 ng/g w.w.), while BzP-2 was detected only in an adult female (2.17 ng/g w.w.). No statistical differences in BzP concentrations were observed between sex and age classes of the white-tailed eagles from Smøla. The occurrence pattern for BzPs was similar to that found in the liver of white-tailed eagles from West Greenland, where BzP-1 (0.71–4.30 ng/g w.w.) and 4-OH-BzP (8.21 and 9.70 ng/g w.w.) were the most detected analogues [30]. BzP-3 was detected in 5 out of 44 samples in the liver of white-tailed eagles from West Greenland, while it was not detected in the current samples from Norway. However, it is noteworthy that BzP-1, 4-OH-BzP, BzP-2 and BzP-8 are documented metabolites of BzP-3 [30], and all of them were found in 17 out of 38 liver samples from Smøla. In a previous study, BzP-1, BzP-3, 4-OH-BzP, and 4,4′-dihydroxybenzophenone (4 DHB; which is an isomer of BzP-1) were reported in 39 unhatched raptor eggs (of 7 wild living species) from Doñana, southwestern Spain, where BzP-1 (range: 23.3−677 ng/g d.w.) and 4-OH-BzP (range: 12.0–3488 ng/g d.w.) were detected in the highest concentrations from all target analytes [50]. BzP-3 was also present in the raptor eggs, but in significantly lower concentrations (range: 18.2–49.3 ng/g d.w.), while 4 DHB was found only in two samples (29.4 and 132 ng/g d.w.) [50].

## 4. Conclusions

In this study, we measured BPs and BzPs in the liver of white-tailed eagles (*Haliaeetus albicilla*) from Smøla, Norway. To the best of our knowledge, this is the first report in raptors from Northern Europe to document multiresidues of both contaminant classes, BPs and BzPs, in raptor livers. From the 38 liver samples analyzed here, the most ubiquitous contaminant was BPAF, with concentrations ranging from 1.08 to 6.68 ng/g w.w., followed by BzP-1, with concentrations ranging from 2.07 to 7.94 ng/g w.w. The fact that BPAF was detected for the first time in all samples implies that this compound reaches remote areas and is readily bioaccumulated in white-tailed eagles and possibly other wildlife. BPAF, which is used in high quantities in the polymer, plastic, and rubber industries, is therefore recommended for further investigation for endocrine disruption effects in raptors. The accumulation of BPs and BzPs in liver tissue from white-tailed eagles warrants further research for potential effects on individual health, physiology, and reproduction in raptors, which has not yet been explored.

## Figures and Tables

**Table 1:** Recoveries (%R) of the target analytes (fortified amounts: 2.5, 10, 20 and 50 ng; *n* = 4 replicates for each amount).

Target Analytes	Amount (ng)	% R (*n* = 4)	RSD % (*n* = 4)
**BPS**	2.5	not quantifiable	-
	10	104	7.5
	20	97	4.8
	50	85	5.5
**BzP-2**	2.5	39	7.1
	10	42	6.8
	20	40	5.9
	50	40	7.4
**4-OH-BzP**	2.5	72	5.2
	10	79	19
	20	82	25
	50	80	31
**BzP-1**	2.5	55	15
	10	44	16
	20	44	28
	50	39	34
**BPF**	2.5	65	3.7
	10	60	14
	20	57	7.4
	50	57	13
**BzP-8**	2.5	57	24
	10	84	18
	20	59	11
	50	64	14
**BPAF**	2.5	104	4.2
	10	98	7.9
	20	98	2.0
	50	94	6.7
**BPA**	2.5	61	12
	10	52	7.0
	20	54	6.0
	50	52	4.5
**BPB**	2.5	38	15
	10	55	27
	20	52	22
	50	62	8.6
**BPM/BPP**	2.5	67	12
	10	83	17
	20	88	9.6
	50	91	14

**Table 2:** Concentrations in ng/g w.w. of the detected target compounds in the livers from the white-tailed eagles (*Haliaeetus albicilla*) (*n* = 38).

Sample Code	Sampling Year	Gender	Estimated Age	BzP-2	4-OH-BzP	BzP-1	BzP-8	BPAF	BPA
HA 07	2006	F	Adult	2.17	0.38	-	-	1.54	-
HA 08	2006	M	Adult	- ^#^	-	-	-	2.22	-
HA 11	2006	F	Subadult	-	0.64	-	-	-	-
HA 14	2008	M	Adult	-	-	2.45	-	-	-
HA 15A	2008	M	Adult	-	-	3.14	-	2.21	-
HA 20	2008	M	Adult	-	-	-	-	5.19	33.8
HA 21	2008	M	Adult	-	0.21	-	-	2.90	-
HA 22A	2009	M	Subadult	-	-	-	-	2.75	-
HA 25	2009	F	Subadult	-	-	2.14	10.5	6.39	-
HA 26	2009	M	Subadult	-	-	-	-	4.98	7.43
117862	2010	M	Juvenile	-	-	-	-	4.54	-
HA 29	2010	F	Adult	-	-	-	-	2.34	3.74
HA 30	2010	M	Subadult	-	2.08	-	-	-	-
HA 31	2010	F	Adult	-	0.14	-	-	2.57	3.36
HA 32	2010	M	Subadult	-	0.39	-	-	-	-
HA 35	2010	F	Subadult	-	-	-	-	6.68	15.1
HA 40	2011	M	Adult	-	-	3.04	-	3.11	-
HA 41	2011	F	Adult	-	0.22	7.94	-	1.08	-
HA 42	2011	M	Adult	-	-	-	-	2.48	-
HA 45	2012	M	Subadult	-	-	-	2.08	1.88	-
HA 46	2012	M	Adult	-	-	-	-	3.08	-
172013	2013	M	Nestling	-	-	-	-	-	3.76
HA 52	2013	F	Subadult	-	-	-	-	2.23	-
23042014	2014	F	Adult	-	-	-	-	1.12	-
HA 56	2014	M	Subadult	-	-	-	-	2.15	10.7
HA 58	2014	F	Subadult	-	-	-	-	1.79	-
HA 59	2014	F	Adult	-	-	3.18	-	3.38	-
HA 60	2015	M	Subadult	-	-	-	-	1.7	-
HA 61	2015	M	Subadult	-	-	-	-	1.98	-
HA 62	2015	F	Adult	-	-	2.74	-	3.12	-
HA 65	2016	M	Subadult	-	-	2.07	-	2.99	-
HA 67	2016	F	Adult	-	-	-	-	3.73	-
HA 68	2016	F	Subadult	-	-	2.47	-	2.33	5.3
HA 72	2016	F	Adult	-	-	-	-	3.28	-
HA 83	2017	M	Adult	-	-	-	-	2.56	-
HA 81	2018	M	Adult	-	0.9	3.25	-	2.32	-
HA 85	2018	F	Adult	-	-	-	-	-	-
HA 88	2018	F	Adult	-	-	-	-	2.25	-
Detection rate				1/38	8/38	10/38	2/38	32/38	8/38
Median *				2.17	0.38	2.89	6.32	2.52	6.37
Mean *				2.17	0.62	3.24	6.32	2.91	10.4
SD *				n.c.	0.60	1.62	4.23	1.33	9.63

- Not found; ^#^ Values < MDLs.; * Values > MDLs were used for the calculation.; n.c.: not calculated.

## Data Availability

The data presented in this study are available in supplementary material.

## Supplementary Materials

The following are available online at https://www.mdpi.com/2305-6304/9/2/34/s1: details on the UPLC-MS/MS analysis and method performance; Table S1: Studied BPs and BzPs with their abbreviation, CAS number, molecular structure, molecular weight (g/mol), octanol-water partition coefficient (log Kow), solubility (25 °C; mg/L) and bioaccumulation factor; Table S2: List of samples with their code, year of sampling, sex and approximation of age of the individuals; Table S3: Bioanalytical method performance characteristics; Table S4: Precursor ions and transitions of the target analytes and internal standards, their retention times, collision energies and cone voltage values for UPLC-MS/MS analysis; Table S5: Recoveries (%R) of the target analytes (post-extraction fortified amounts: 2.5, 10, 20 and 50 ng; *n* = 2 replicates for each amount). Table S6: Concentrations in ng/g w.w. of the detected target compounds in the livers from the white-tailed eagles sorted by sex and estimated age; Table S7: Concentrations in ng/g w.w. of the detected target compounds in the livers from the white-tailed eagles sorted by sampling year.
