# Supplementary material for: Occurrence of Bisphenols and Benzophenone UV Filters in White-Tailed Eagles (Haliaeetus albicilla) from Smøla, Norway
Source: Toxics. 2021 Feb 9;9(2):34. doi: 10.3390/toxics9020034 (PMC7914477; doi:10.3390/toxics9020034)
Supplement: Supplementary file 1 [file toxics-09-00034-s001.pdf]

Supplementary Materials: Occurrence of Bisphenols and Benzophenone UV Filters in White-Tailed Eagles (*Haliaeetus albicilla*) from Smøla, Norway

Bernat Oró-Nolla, Silvia Lacorte, Kristine Vike-Jonas, Susana V. Gonzalez, Torgeir Nygård, Alexandros G. Asimakopoulos and Veerle L.B. Jaspers

1. Physicochemical Properties of Studied BPs and BzPs

All the studied BPs and BzPs with their abbreviation, CAS number, molecular structure, molecular weight (g/mol), octanol-water partition coefficient (log K_{ow}), solubility (25 °C; mg/L) and bioaccumulation factor are listed in **Table S1**.

2. UPLC-MS/MS Analysis

The chromatographic separation was carried out using an Acquity UPLC I-Class system (Waters, Milford, MA, USA) coupled to a triple quadrupole mass analyser (QqQ; Xevo TQ-S)with a ZSpray ESI ion source (Waters, Milford, MA, USA). The LC column used was a Kinetex C18 (50 × 2.1mm, 1.3 µm) connected to a Phenomenex C18 guard column (2.0 × 2.1mm). The column temperature was set at 30 °C. The mobile phase consisted of solvent (A) 0.1% *v/v* ammonium hydroxide in Milli-Q water and (B)methanol. The flowrate was 300 µLmin−1 and the injection volume were 4 µL. The gradient elution initiated with 75% A, held for 10 s, decreased to 25% A within 3.4 min, then further decreased to 1% A, held for 30 s, and reverted to 75% A that was held for 20 s, for a total time run of 4 min. The mass spectrometer was operated in multiple reaction monitoring (MRM) mode. Electrospray ionization was performed under negative ionization mode (ESI−). Optimal source settings were the following:source gas temperature 150 °C, capillary voltage −1500 V and nebulizer gas pressure 7.0 bar. Quantification of the target analytes was accomplished based on the internal standard method and with matrix-matched calibration standards [1], [2].

3. Method Performance

Correlation coefficients were assessed by running calibration curves prepared in methanol (fortified with target BPs and BzPs at 0.1, 0.2, 0.5, 1, 2, 5, 10, 20 and 50 ng/mL and ISs at 20 ng/mL). The correlation coefficients in all cases were above 0.98 **(Table S3)**. Precursor and product ions, retention times (RT) and relevant detection UPLC-MS/MS parameters of each target analyte and IS are presented in **Table S4**. The isomers, BPM and BPP, were quantified in samples as a single 1:1 mixture [3]. The method detection limits were ranging from 0.04 to 2.92 ng/g w.w. **(Table S3)**. The precision of the UPLC-MS/MS method was evaluated in terms of repeatability expressed as relative standard deviation (RSD%) and for most target analytes was < 15% in the liver; overall ranged from 2.94 to 15.9% **(Table S3)**. The instrumental repeatability was assessed by consecutive injections of standard solutions at an amount of 10 ng (n = 5, k = 1 day). The instrumental reproducibility was assessed by consecutive injections of standard solutions at 2.5 (n = 5) and at 20 ng (n = 5) in-between two days (k = 2) **(Table S3)**. The matrix effects (%) for the target analytes are shown in **Table S3**. All target analytes demonstrated ionization suppression with the highest observed for BPAF and BPF, except for BzP-8 and BPM/BPP, which demonstrated slight signal enhancement.

Table S1. Studied BPs and BzPs with their abbreviation, CAS number, molecular structure, molecular weight (g/mol), octanol-water partition coefficient (log K_{ow}), solubility (25 °C; mg/L) and bioaccumulation factor.

Target Analytes	Molecular Structure	Molecular Weight / g/mol	log K_{ow}	Solubility (25 °C) / mg/L	Bioaccumulation Factor (log BAF) [f]

**Bisphenol S (BPS)**<br>4,4′-sulfonyldiphenol<br>CAS: 80-09-1	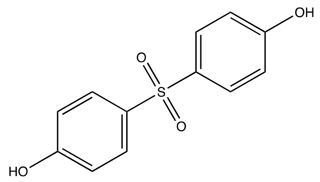	250.3	1.65 [b]	3518	0.548
**Benzophenone-2 (BzP-2)**<br>2,2′,4,4′-tetrahydroxybenzophenone<br>CAS: 131-55-5	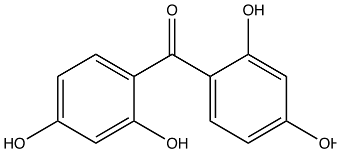	246.2	3.16 [c]	398.5	0.614
**4-hydroxybenzophenone (4-OH-BzP)**<br>CAS: 1137–42–4	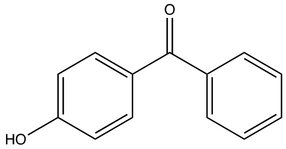	198.2	3.07 [d]	405.8	1.619
**Benzophenone-1 (BzP-1)**<br>2,4-dihydroxybenzophenone<br>CAS: 131–56–6	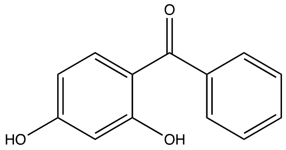	214.2	3.17 [c]	413.4	0.964
**Bisphenol F (BPF)**<br>4,4′-dihydroxydiphenylmethane<br>CAS: 620-92-8	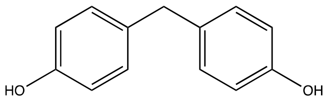	200.2	2.90 [a]	542.8	1.448
**Benzophenone-8 (BzP-8)**<br>2,2′-dihydroxy-4-methoxybenzophenone<br>CAS: 131-53-3	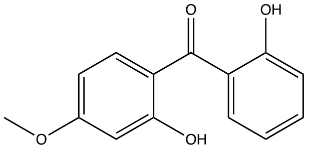	244.2	3.93 [c]	52.73	1.673
**Bisphenol AF (BPAF)**<br>4,4′-(hexafluoroisopropylidene) diphenol<br>CAS: 1478-61-1	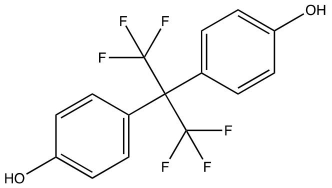	336.2	5.50 [a]	4.302	2.808
**Bisphenol A (BPA)**<br>2,2-bis(4-hydroxyphenyl) propane<br>CAS: 80-05-7	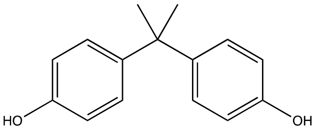	228.3	3.60 [a]	172.7	2.238
**Bisphenol B (BPB)**<br>2,2-bis(4-hydroxyphenyl) butane<br>CAS: 77-40-7	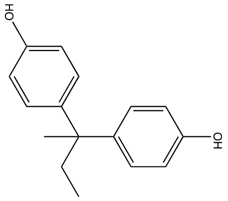	242.3	4.20 [a]	29.23	2.231
**Bisphenol M (BPM)**<br>1,3-bis(2-(4-hydroxyphenyl)-2-propyl) benzene<br>CAS: 13595-25-0	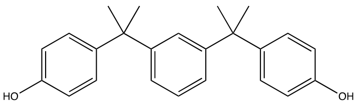	346.5	6.25[e]	0.113	3.958
**Bisphenol P (BPP)**<br>4,4′-(1,4-phenylenediisopropylidene) bisphenol<br>CAS: 2167-51-3	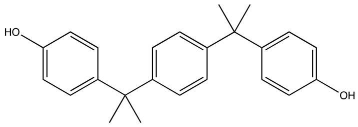	346.5	6.25[e]	0.113	3.958

[a] from [4]; [b] from [5]; [c] from [6], [d] from [7]; [e] from [8]; and [f] from [9].

Table S2. List of samples with their code, year of sampling, sex and approximation of age of the individuals.

Sample Code	Year	Gender	Estimated Age
**HA 07**	2006	Female	Adult
**HA 08**	2006	Male	Adult
**HA 11**	2006	Female	Subadult
**HA 14**	2008	Male	Adult
**HA 15A**	2008	Male	Adult
**HA 20**	2008	Male	Adult
**HA 21**	2008	Male	Adult
**HA 22A**	2009	Male	Subadult
**HA 25**	2009	Female	Subadult
**HA 26**	2009	Male	Subadult
**117862**	2010	Male	Juvenile
**HA 29**	2010	Female	Adult
**HA 30**	2010	Male	Subadult
**HA 31**	2010	Female	Adult
**HA 32**	2010	Male	Subadult
**HA 35**	2010	Female	Subadult
**HA 40**	2011	Male	Adult
**HA 41**	2011	Female	Adult
**HA 42**	2011	Male	Adult
**HA 45**	2012	Male	Subadult
**HA 46**	2012	Male	Adult
**172013**	2013	Male	Nestling
**HA 52**	2013	Female	Subadult
**23042014**	2014	Female	Adult
**HA 56**	2014	Male	Subadult
**HA 58**	2014	Female	Subadult
**HA 59**	2014	Female	Adult
**HA 60**	2015	Male	Subadult
**HA 61**	2015	Male	Subadult
**HA 62**	2015	Female	Adult
**HA 65**	2016	Male	Subadult
**HA 67**	2016	Female	Adult
**HA 68**	2016	Female	Subadult
**HA 72**	2016	Female	Adult
**HA 83**	2017	Male	Adult
**HA 81**	2018	Male	Adult
**HA 85**	2018	Female	Adult
**HA 88**	2018	Female	Adult

Table S3. Bioanalytical method performance characteristics.

Target Analytes	Calibration Curves		Precision %	Repeatability %	Reproducibility		Detection Limits			Matrix Effects %
	R^2	Linearity (ng·mL^{-1})	10 ng (n = 3)	10 ng (n = 5)	2.5 ng (n = 5)	20 ng (n = 5)	IDL (ng/mL)	MDL (ng/g)	RSD %	
**BPS**	0.998	0.2–50	3.67	1.7	11	13	0.12	0.27	14	−39
**BzP-2**	0.9993	0.2–50	6.29	3.6	9.9	5.8	0.14	0.19	7.1	−54
**4-OH-BzP**	0.996	0.1–50	13.1	4.1	17	23	0.02	0.05	5.2	−20
**BzP-1**	0.998	0.1–50	8.04	5.6	24	19	0.01	0.04	15	−42
**BPF**	0.98	0.2–50	10.4	11	33	14	0.01	2.92	3.8	−1.9
**BzP-8**	0.9994	0.1–50	13.0	12	39	6.9	0.06	1.18	23	13
**BPAF**	0.9996	0.1–50	4.3	9.5	11	3.0	0.04	0.86	4.2	−0.8
**BPA**	0.9993	0.1–50	2.94	9.5	36	7.2	0.04	2.73	12	−20
**BPB**	0.9986	0.2–50	15.9	11	21	6.0	0.13	1.47	15	−75
**BPM/BPP**	0.9998	0.1–50	6.54	8.3	31	24	0.04	0.16	14	15

Table S4. Precursor ions and transitions of the target analytes and internal standards, their retention times, collision energies and cone voltage values for UPLC-MS/MS analysis.

Target Analytes	Retention Time (min)	Precursor Ion (m/z)	Cone Voltage (V)	Quantification Transition (m/z)	Collision Energy (eV)	Confirmation Transition (m/z)	Collision Energy (eV)	Internal Standards Used
**BPS**	0.38	249 [M-H]-	40	249 > 108	26	249 > 156	22	BPS (^{13}C)
**BzP-2**	0.39	245 [M-H]-	40	245 >109	16	245 > 135	14	BPS (^{13}C)
**4-OH-BzP**	0.48	197 [M-H]-	60	197 > 92	28	197 > 120	22	BPS (^{13}C)
**BzP-1**	0.48	213 [M-H]-	46	213 > 135	18	213 > 91	28	BPS (^{13}C)
**BPF**	1.81	199 [M-H]-	46	199 > 93	20	199 > 105	20	BPF (^{13}C)
**BzP-8**	1.91	243 [M-H]-	46	243 > 123	16	243 > 93	18	BPA (^{13}C)
**BPAF**	2.04	355 [M-H]-	40	355 > 265	24	355 > 177	42	BPAF (^{13}C)
**BPA**	2.36	227 [M-H]-	50	227 > 212	18	227 > 133	24	BPA (^{13}C)
**BPB**	2.71	241 [M-H]-	20	241 > 212	18	--	--	BPB (^{13}C)
**BPM/BPP**	3.95	345 [M-H]-	14	345 > 330	26	345 > 133	46	BPA (^{13}C)
**BPS (^{13}C)**	0.39	261 [M-H]-	50	261 > 114	28	261 > 162	20	
**BPF (^{13}C)**	1.81	211 [M-H]-	22	211 > 99	24	211 > 111	26	
**BPAF (^{13}C)**	2.04	347 [M-H]-	20	347 > 277	24	347 > 208	36	
**BPA (^{13}C)**	2.36	239 [M-H]-	30	239 > 224	20	--	--	
**BPB (^{13}C)**	2.71	253 [M-H]-	16	253 > 224	18	--	--	

-- **Not considered values.**

Table S5. Recoveries (%R) of the target analytes (post-extraction fortified amounts: 2.5, 10, 20 and 50 ng; *n* = 2 replicates for each amount).

Target Analytes	Amount (ng)	Average (*n* = 2)	RSD % (*n* = 2)
**BPS**	2.5	not quantifiable	-
	10	100	5.0
	20	90	8.4
	50	84	5.5
**BzP-2**	2.5	62	5.3
	10	64	6.6
	20	65	2.7
	50	64	0.7
**4-OH-BzP**	2.5	120	23
	10	123	20
	20	117	9.4
	50	125	3.8
**BzP-1**	2.5	89	8.2
	10	86	9.9
	20	86	11
	50	89	17
**BPF**	2.5	53	10
	10	56	2.1
	20	54	6.6
	50	54	12
**BzP-8**	2.5	69	6.1
	10	72	8.0
	20	73	6.1
	50	70	3.9
**BPAF**	2.5	84	14.4
	10	95	2.1
	20	94	4.6

	50	91	6.3
**BPA**	2.5	46	13
	10	53	4.4
	20	53	3.0
	50	52	2.9
**BPB**	2.5	49	23
	10	55	15
	20	56	16
	50	53	17
**BPM/BPP**	2.5	70	5.7
	10	75	4.3
	20	75	3.7
	50	73	4.3

Table S6. Concentrations in ng/g w.w. of the detected target compounds in the livers from the white-tailed eagles (*Haliaeetus albicilla*) (*N* = 38); sorted by sex and estimated age.

	BzP-2	4-OH-BzP	BzP-1	BzP-8	BPAF	BPA
**Females (*n* = 17)**						
Detection rate	1/17	4/17	5/17	1/17	15/17	4/17
Median (ng/g w.w.)*	2.17	0.31	2.74	10.55	2.34	4.52
Mean (ng/g w.w.)*	2.17	0.35	3.69	10.55	2.92	6.86
SD*	n.c.	0.19	2.15	n.c.	1.61	4.78
RSD %*	n.c.	55.4	58.2	n.c.	55.0	69.7
**Males (*n* = 21)**						
Detection rate	0/21	4/21	5/21	1/21	17/21	4/21
Median (ng/g w.w.)*	n.c.	0.65	3.04	2.08	2.56	9.07
Mean (ng/g w.w.)*	n.c.	0.90	2.79	2.08	2.88	13.93
SD*	n.c.	0.73	0.45	0.00	1.02	11.74
RSD %*	n.c.	81.5	16.3	0.00	35.5	84.3
**Adults (*n* = 21)**						
Detection rate	1/21	5/21	7/21	0/21	19/21	3/21
Median (ng/g w.w.)*	2.17	0.22	3.14	n.c.	2.56	3.74
Mean (ng/g w.w.)*	2.17	0.37	3.68	n.c.	2.66	13.63
SD*	0.00	0.28	1.76	n.c.	0.92	14.26
RSD %*	0.00	74.7	47.9	n.c.	34.6	104.6
**Sub-adults (*n* = 15)**						
Detection rate	0/15	3/15	3/15	2/15	12/15	4/15
Median (ng/g w.w.)*	n.c.	0.64	2.14	6.32	2.28	9.07
Mean (ng/g w.w.)*	n.c.	1.04	2.23	6.32	3.15	9.62
SD*	n.c.	0.74	0.17	4.24	1.73	3.68
RSD %*	n.c.	71.8	7.8	67.1	54.9	38.2
**Juvenile and nestling (*n* = 2)**						
Detection rate	0/2	0/2	0/2	0/2	1/2	1/2
Median (ng/g w.w.)*	n.c.	n.c.	n.c.	n.c.	4.54	3.76
Mean (ng/g w.w.)*	n.c.	n.c.	n.c.	n.c.	4.54	3.76
SD*	n.c.	n.c.	n.c.	n.c.	n.c.	n.c.
RSD %*	n.c.	n.c.	n.c.	n.c.	n.c.	n.c.

* Values > MDLs were used for the calculation.; n.c.: not calculated.

Table S7. Concentrations in ng/g w.w. of the detected target compounds in the livers from the white-tailed eagles (*Haliaeetus albicilla*) (*N* = 38); sorted by sampling year.

Sample Code	Year	Gender	Estimated Age	BzP-2	4-OH-BzP	BzP-1	BzP-8	BPAF	BPA
**HA 07**	2006	F	Adult	2.17	0.38	-	-	1.54	-
**HA 08**	2006	M	Adult	-	-	-	-	2.22	-

**HA 11**	2006	F	Subadult	-	0.64	-	-	-	-
Detection rate				1/3	2/3	0/3	0/3	2/3	0/3
Median (ng/g w.w.)*				2.17	0.51	n.c.	n.c.	1.88	n.c.
Mean (ng/g w.w.)*				2.17	0.51	n.c.	n.c.	1.88	n.c.
SD*				n.c.	0.13	n.c.	n.c.	0.34	n.c.
RSD %*				n.c.	25.5	n.c.	n.c.	18.1	n.c.
**HA 14**	2008	M	Adult	-	-	2.45	-	-	-
**HA 15A**	2008	M	Adult	-	-	3.14	-	2.21	-
**HA 20**	2008	M	Adult	-	-	-	-	5.19	33.8
**HA 21**	2008	M	Adult	-	0.21	-	-	2.90	-
Detection rate				0/4	1/4	2/4	0/4	3/4	1/4
Median (ng/g w.w.)*				n.c.	0.21	2.80	n.c.	2.90	33.8
Mean (ng/g w.w.)*				n.c.	0.21	2.80	n.c.	3.43	33.8
SD*				n.c.	n.c.	0.35	n.c.	1.27	n.c.
RSD %*				n.c.	n.c.	12.3	n.c.	37.1	n.c.
**HA 22A**	2009	M	Subadult	-	-	-	-	2.75	-
**HA 25**	2009	F	Subadult	-	-	2.14	10.5	6.39	-
**HA 26**	2009	M	Subadult	-	-	-	-	4.98	7.43
Detection rate				0/3	0/3	1/3	1/3	3/3	1/3
Median (ng/g w.w.)*				n.c.	n.c.	2.14	10.55	4.98	7.43
Mean (ng/g w.w.)*				n.c.	n.c.	2.14	10.55	4.71	7.43
SD*				n.c.	n.c.	n.c.	n.c.	1.50	n.c.
RSD %*				n.c.	n.c.	n.c.	n.c.	31.8	n.c.
**117862**	2010	M	Juvenile	-	-	-	-	4.54	-
**HA 29**	2010	F	Adult	-	-	-	-	2.34	3.74
**HA 30**	2010	M	Subadult	-	2.08	-	-	-	-
**HA 31**	2010	F	Adult	-	0.14	-	-	2.57	3.36
**HA 32**	2010	M	Subadult	-	0.39	-	-	-	-
**HA 35**	2010	F	Subadult	-	-	-	-	6.68	15.0
Detection rate				0/6	3/6	0/6	0/6	4/6	3/6
Median (ng/g w.w.)*				n.c.	0.39	n.c.	n.c.	3.56	3.74
Mean (ng/g w.w.)*				n.c.	0.87	n.c.	n.c.	4.03	7.38
SD*				n.c.	0.86	n.c.	n.c.	1.75	5.42
RSD %*				n.c.	99.0	n.c.	n.c.	43.4	73.4
**HA 40**	2011	M	Adult	-	-	3.04	-	3.11	-
**HA 41**	2011	F	Adult	-	0.22	7.94	-	1.08	-
**HA 42**	2011	M	Adult	-	-	-	-	2.48	-
Detection rate				0/3	1/3	2/3	0/3	3/3	0/3
Median (ng/g w.w.)*				n.c.	0.22	5.49	n.c.	2.48	n.c.
Mean (ng/g w.w.)*				n.c.	0.22	5.49	n.c.	2.22	n.c.
SD*				n.c.	n.c.	2.45	n.c.	0.85	n.c.
RSD %*				n.c.	n.c.	44.6	n.c.	38.2	n.c.
**HA 45**	2012	M	Subadult	-	-	-	2.08	1.88	-
**HA 46**	2012	M	Adult	-	-	-	-	3.08	-
Detection rate				0/2	0/2	0/2	1/2	2/2	0/2
Median (ng/g w.w.)*				n.c.	n.c.	n.c.	2.08	2.48	n.c.
Mean (ng/g w.w.)*				n.c.	n.c.	n.c.	2.08	2.48	n.c.
SD*				n.c.	n.c.	n.c.	n.c.	0.60	n.c.
RSD %*				n.c.	n.c.	n.c.	n.c.	24.1	n.c.

**172013**	2013	M	Nestling	-	-	-	-	-	3.76
**HA 52**	2013	F	Subadult	-	-	-	-	2.23	-
Detection rate				0/2	0/2	0/2	0/2	1/2	1/2
Median (ng/g w.w.)*				n.c.	n.c.	n.c.	n.c.	2.23	3.76
Mean (ng/g w.w.)*				n.c.	n.c.	n.c.	n.c.	2.23	3.76
SD*				n.c.	n.c.	n.c.	n.c.	n.c.	n.c.
RSD %*				n.c.	n.c.	n.c.	n.c.	n.c.	n.c.
**23042014**	2014	F	Adult	-	-	-	-	1.12	-
**HA 56**	2014	M	Subadult	-	-	-	-	2.15	10.7
**HA 58**	2014	F	Subadult	-	-	-	-	1.79	-
**HA 59**	2014	F	Adult	-	-	3.18	-	3.38	-
Detection rate				0/4	0/4	1/4	0/4	4/4	0.25
Median (ng/g w.w.)*				n.c.	n.c.	3.18	n.c.	1.97	10.7
Mean (ng/g w.w.)*				n.c.	n.c.	3.18	n.c.	2.11	10.7
SD*				n.c.	n.c.	n.c.	n.c.	0.82	n.c.
RSD %*				n.c.	n.c.	n.c.	n.c.	38.9	n.c.
**HA 60**	2015	M	Subadult	-	-	-	-	1.70	-
**HA 61**	2015	M	Subadult	-	-	-	-	1.98	-
**HA 62**	2015	F	Adult	-	-	2.74	-	3.12	-
Detection rate				0/3	0/3	1/3	0/3	3/3	0/3
Median (ng/g w.w.)*				n.c.	n.c.	2.74	n.c.	1.98	n.c.
Mean (ng/g w.w.)*				n.c.	n.c.	2.74	n.c.	2.27	n.c.
SD*				n.c.	n.c.	n.c.	n.c.	0.61	n.c.
RSD %*				n.c.	n.c.	n.c.	n.c.	27.1	n.c.
**HA 65**	2016	M	Subadult	-	-	2.07	-	2.99	-
**HA 67**	2016	F	Adult	-	-	-	-	3.73	-
**HA 68**	2016	F	Subadult	-	-	2.47	-	2.33	5.30
**HA 72**	2016	F	Adult	-	-	-	-	3.28	-
Detection rate				0/4	0/4	2/4	0/4	4/4	1/4
Median (ng/g w.w.)*				n.c.	n.c.	2.27	n.c.	3.14	5.30
Mean (ng/g w.w.)*				n.c.	n.c.	2.27	n.c.	3.08	5.30
SD*				n.c.	n.c.	0.20	n.c.	0.51	n.c.
RSD %*				n.c.	n.c.	8.81	n.c.	16.4	n.c.
**HA 83**	2017	M	Adult	-	-	-	-	2.56	-
Detection rate				0/1	0/1	0/1	0/1	1/1	0/1
Median (ng/g w.w.)*				n.c.	n.c.	n.c.	n.c.	2.56	n.c.
Mean (ng/g w.w.)*				n.c.	n.c.	n.c.	n.c.	2.56	n.c.
SD*				n.c.	n.c.	n.c.	n.c.	n.c.	n.c.
RSD %*				n.c.	n.c.	n.c.	n.c.	n.c.	n.c.
**HA 81**	2018	M	Adult	-	0.90	3.25	-	2.32	-
**HA 85**	2018	F	Adult	-	-	-	-	-	-
**HA 88**	2018	F	Adult	-	-	-	-	2.25	-
Detection rate				0/3	1/3	1/3	0/3	2/3	0/3
Median (ng/g w.w.)*				n.c.	0.90	3.25	n.c.	2.29	n.c.
Mean (ng/g w.w.)*				n.c.	0.90	3.25	n.c.	2.29	n.c.
SD*				n.c.	n.c.	n.c.	n.c.	0.03	n.c.
RSD %*				n.c.	n.c.	n.c.	n.c.	1.53	n.c.

#Values < MDLs.; *Values > MDLs were used for the calculation.; n.c.: not calculated.

References

1. Asimakopoulos, A.G.; Wang, L.; Thomaidis, N.S.; Kannan, K. A multi-class bioanalytical methodology for the determination of bisphenol A diglycidyl ethers, p-hydroxybenzoic acid esters, benzophenone-type ultraviolet filters, triclosan, and triclocarban in human urine by liquid chromatography–tandem mass spectrometry. *J. Chromatogr. A* **2014**, *1324*, 141–148, doi:10.1016/j.chroma.2013.11.031.
2. Asimakopoulos, A.G.; Elangovan, M.; Kannan, K. Migration of parabens, bisphenols, benzophenone-type UV filters, triclosan, and triclocarban from teethers and its implications for infant exposure. *Environ. Sci. Technol.* **2016**, *50*, 13539–13547, doi:10.1021/acs.est.6b04128.
3. González-Rubio, S.; Vike-Jonas, K.; Gonzalez, S.V.; Ballesteros-Gomez, A.; Sonne, C.; Dietz, R.; Boertmann, D.; Rasmussen, L.M.; Jaspers, V.L.B.; Asimakopoulos, A.G. Bioaccumulation potential of bisphenols and benzophenone UV filters: A multiresidue approach in raptor tissues. *Sci. Total. Environ.* **2020**, *741*, 140330, doi:10.1016/j.scitotenv.2020.140330.
4. Yang, Y.; Lu, L.; Zhang, J.; Yang, Y.; Wu, Y.; Shao, B. Simultaneous determination of seven bisphenols in environmental water and solid samples by liquid chromatography–electrospray tandem mass spectrometry. *J. Chromatogr. A* **2014**, *1328*, 26–34, doi:10.1016/j.chroma.2013.12.074.
5. Yan, Z.; Liu, Y.; Yan, K.; Wu, S.; Han, Z.; Guo, R.; Chen, M.; Yang, Q.; Zhang, S.; Chen, J. Bisphenol analogues in surface water and sediment from the shallow Chinese freshwater lakes: Occurrence, distribution, source apportionment, and ecological and human health risk. *Chemosphere* **2017**, *184*, 318–328, doi:10.1016/j.chemosphere.2017.06.010.
6. Negreira, N.; Rodríguez, I.; Ramil, M.; Rubi, E.; Cela, R.; Pereiro, I.R. Solid-phase extraction followed by liquid chromatography-tandem mass spectrometry for the determination of hydroxylated benzophenone UV absorbers in environmental water samples. *Anal. Chim. Acta* **2009**, *654*, 162–170, doi:10.1016/j.aca.2009.09.033.
7. Zhang, Z.; Ren, N.; Li, Y.-F.; Kunisue, T.; Gao, D.; Kannan, K. Determination of benzotriazole and benzophenone UV Filters in sediment and sewage sludge. *Environ. Sci. Technol.* **2011**, *45*, 3909–3916, doi:10.1021/es2004057.
8. United States Environmental Protection Agency (US EPA). 2020. *Estimation Programs Interface SuiteTM (version 4.11) for Microsoft® Windows*; Washington, US EPA.
9. United States Environmental Protection Agency (US EPA). 2020. *BAF Arnot-Gobas Method. Estimation Programs Interface SuiteTM (version 4.11) for Microsoft® Windows*; Washington, US EPA.
